# Progestins Upregulate FKBP51 Expression in Human Endometrial Stromal Cells to Induce Functional Progesterone and Glucocorticoid Withdrawal: Implications for Contraceptive- Associated Abnormal Uterine Bleeding

**DOI:** 10.1371/journal.pone.0137855

**Published:** 2015-10-05

**Authors:** Ozlem Guzeloglu Kayisli, Umit A. Kayisli, Murat Basar, Nihan Semerci, Frederick Schatz, Charles J. Lockwood

**Affiliations:** 1 Department of Obstetrics and Gynecology, Morsani College of Medicine, University of South Florida, Tampa, Florida, United State of America; 2 Department of Obstetrics and Gynecology, School of Medicine, Yale University, New Haven, Connecticut, United State of America; University of Edinburgh, UNITED KINGDOM

## Abstract

Use of long-acting progestin only contraceptives (LAPCs) offers a discrete and highly effective family planning method. Abnormal uterine bleeding (AUB) is the major side effect of, and cause for, discontinuation of LAPCs. The endometria of LAPC-treated women display abnormally enlarged, fragile blood vessels, decreased endometrial blood flow and oxidative stress. To understanding to mechanisms underlying AUB, we propose to identify LAPC-modulated unique gene cluster(s) in human endometrial stromal cells (HESCs). Protein and RNA isolated from cultured HESCs treated 7 days with estradiol (E_2_) or E_2_+ medroxyprogesterone acetate (MPA) or E_2_+ etonogestrel (ETO) or E_2_+ progesterone (P4) were analyzed by quantitative Real-time (q)-PCR and immunoblotting. HSCORES were determined for immunostained-paired endometria of pre-and 3 months post-Depot MPA (DMPA) treated women and ovariectomized guinea pigs (GPs) treated with placebo or E_2_ or MPA or E_2_+MPA for 21 days. In HESCs, whole genome analysis identified a 67 gene group regulated by all three progestins, whereas a 235 gene group was regulated by E_2_+ETO and E_2_+MPA, but not E_2_+P4. Ingenuity pathway analysis identified glucocorticoid receptor (GR) activation as one of upstream regulators of the 235 MPA and ETO-specific genes. Among these, microarray results demonstrated significant enhancement of FKBP51, a repressor of PR/GR transcriptional activity, by both MPA and ETO. q-PCR and immunoblot analysis confirmed the microarray results. In endometria of post-DMPA *versus* pre-DMPA administered women, FKBP51 expression was significantly increased in endometrial stromal and glandular cells. In GPs, E_2_+MPA or MPA significantly increased FKBP51 immunoreactivity in endometrial stromal and glandular cells *versus* placebo- and E_2_-administered groups. MPA or ETO administration activates GR signaling and increases endometrial FKBP51 expression, which could be one of the mechanisms causing AUB by inhibiting PR and GR-mediated transcription. The resultant PR and/or GR-mediated functional withdrawal may contribute to associated endometrial inflammation, aberrant angiogenesis, and bleeding.

## Introduction

Long-acting progestin-only contraceptives (LAPCs) are safe, effective and inexpensive making them particularly ideal for use by women in underdeveloped countries with limited access to medical care [1]. Unlike estrogen-containing contraceptives, these progestins can be used safely during lactation and by women in whom estrogen use is contraindicated [2]. Currently available formulations include the depot form of medroxyprogesterone acetate (DMPA), an injectable form whose action persists for three months; intrauterine systems (IUS) s that release levonorgestrel (LNG) for either three or five years; and a single subdermal implantable rod, which releases etonogestrel (ETO) for three years [3–5].

More than a million unintended pregnancies occur in the U.S. each year due to the misuse or discontinuation of contraceptives [6]. The major reason for discontinuation of LAPC is the occurrence of abnormal uterine bleeding (AUB) [1, 7], which is a source of personal annoyance, discomfort and/or religious and social taboo in specific societies [7, 8]. Unlike menstrual bleeding originating globally from spiral arterioles in response to progesterone (P4) withdrawal, LAPC-associated AUB occurs intermittently and focally from irregularly distributed superficial, abnormally enlarged, fragile microvessels [9, 10]. Microscopic examination of endometrial biopsies from adjacent bleeding (BL) and non-bleeding (NBL) sites in LAPC users revealed that only BL sites displayed these abnormal microvessels enmeshed in a compromised extracellular matrix (ECM). These damaged microvessels are contiguous with decidualized human endometrial stromal cells (HESCs) expressing high levels of tissue factor (TF), the primary initiator of hemostasis via factor VIIa and factor Xa/Va generated thrombin [11, 12]. Thus, LAPC-induced microvascular damage should contribute to excess local thrombin generation by increasing delivery of circulating clotting factors to HESC-derived TF. While thrombin prevents bleeding by activating platelets and generating fibrin, it binds to protease activated receptors (PARs) on human endometrial endothelial cell (HEEC) to increase endothelial permeability [13]. Thrombin also binds HESC-expressed PAR–1 to promote aberrant angiogenesis and inflammation by increasing levels of vascular endothelial growth factor (VEGF) [14] and interleukin–8 (IL–8) [15]. Thrombin also induces HESC-derived matrix metalloproteinase (MMP)-1, which preferentially degrades interstitial collagens, and MMP–3, which, in turn, degrades several other ECM proteins and activates secreted MMP zymogens [13]. These proteolytic and inflammatory processes markedly contribute to LAPC-induced AUB.

The FK506-binding proteins (FKBP51 and FKBP52) belong to a family of immunophilins that mediate the actions of specific immunosuppressive drugs [16, 17]. Two members, FKBP51 and FKBP52, share 70% homology and contain a tetracopeptide repeat domain that binds to the C-terminus of heat shock protein 90 (Hsp–90). The FKBP51 promoter region and introns contain several progesterone or glucocorticoid response elements (PREs or GREs), which mediate transcriptional induction of FKBP51 by progesterone receptor (PR) and/or glucocorticoid receptor (GR) [17]. In turn, elevated levels of FKBP51 can inhibit transcriptional activity of both PR and GR [18–20]. We posit that in HESCs, FKBP51 causes functional P4 and/or glucocorticoid (GC) withdrawal by inhibiting transcriptional activity of PR and/or GR.

The contraceptive action of LAPCs results from inhibition of ovulation, thickening of the cervical mucus and impaired cyclic changes in the functional endometrium that impede implantation [21]. However, the LAPC-induced cellular and molecular changes that trigger AUB are poorly understood. This study hypothesizes that MPA or ETO modulates the expression of unique gene cluster(s) in endometrial cells, which initiate molecular changes that induce AUB. To identify these molecular modifications, we performed whole genome microarray analysis on primary cultures of HESCs treated with progestogens. Comparative analysis determined unique (individual) and common signaling pathways induced by MPA or ETO *vs*. P4. Whole genome analysis of the current study identified FKBP51 as one of genes significantly induced by MPA and ETO, but not P4. Therefore, subsequent experiments evaluated the regulation of FKBP51 in cultured HESCs as well as paired endometrial tissues obtained from women pre- and post-DMPA therapy. Previous studies in our laboratory demonstrated that the guinea pig (GP) is a relevant model with which to evaluate the endometrial effects of LAPCs. Like human endometrium, the GP endometrium displays closely related human features such as spontaneous estrus cycling and hemochorial placentation [22]. Thus, findings obtained from HESC cultures and human tissues were augmented by observations in the endometrium of MPA-treated GPs. In addition given the critical role that thrombin plays in mediating LAPC-induced AUB, we evaluated the effects of FKBP51 on thrombin modulation of inflammation in MPA-treated HESCs.

## Materials and Methods

### Antibodies and Compounds

The FKBP51 goat polyclonal antibody (Cat# AF4094) was obtained from R&D Systems, Minneapolis, MN. The β-actin (Cat# 5125), total and phosphorylated AKT (Cat#4085 and 4060, respectively) and total and phosphorylated ERK1/2 MAPK (Cat# 4695 and 4377, respectively) rabbit monoclonal antibodies were purchased from Cell Signaling Technology, Beverly, MA. The following secondary antibodies were obtained from Vector Labs, Burlingame, CA; peroxidase-conjugated anti-goat (Cat# PI–9500), biotinylated anti-goat (Cat# BA–0500). The following chemicals were obtained from Sigma-Aldrich, St. Louis, MO; estradiol (E_2_, Cat#E8875), progesterone (Cat#P8783) and MPA (Cat#M1629). ETO was purchased from Organon, Roseland, NJ.

### Cell Cultures

Frozen HESCs was acquired from previously banked samples isolated and characterized as previously described [23] from specimens of human endometrium obtained from reproductive age undergoing hysterectomy for benign disease after obtaining informed written consent under approval of the Yale University School of Medicine Human Investigation Committee (HIC#22334) and pending approval of University of South Florida (HIC#Pro00019480). All samples used in our study were de-identified when they were obtained. Thawed HESCs (n = 6) were grown to confluence in basal medium (BM), a phenol red-free 1:1 v/v mix of Dulbecco's MEM/Ham's F–12 (Gibco, Grand Island, NY), with 100U/ml penicillin, 100 μg/ml streptomycin, 0.25 μg/ml fungizone complex (Gibco) supplemented with 10% charcoal-stripped calf serum (Gibco). Confluent HESCs were incubated in parallel in BM with 0.1% ethanol (vehicle control) or 10^−8^ M E_2_ or E_2_+10^−7^ M P_4_ (E_2_+P_4_), or 10^−7^ M of ETO (E_2_+ETO) or 10^−7^ M of MPA (E_2_+MPA). After 7 days, the cultures were washed twice with 1X phosphate-buffered saline (PBS) to remove residual serum, then placed in a serum-free defined media (DM) comprising BM plus ITS^+^ (insulin, transferrin, selenious and linoleic acid) premix (BD Biosciences, Bedford, MA), 5 μM of FeSO_4_, 50 μM of ZnSO_4_, 1 nM of CuSO_4_, 20 nM of Na_2_SeO_3_ trace elements (Sigma-Aldrich), 50 μg/ml of ascorbic acid (Sigma-Aldrich) and 50 ng/ml of recombinant epidermal growth factor (Becton-Dickinson, Bedford, MA) for 24h. The cultures were washed twice with 1X PBS to remove the residual steroids and DM containing corresponding vehicle control or steroids was added to the cultures for 6h or 24h. At the end of incubation periods, HESCs were washed with ice-cold 1X PBS and stored at −70°C until used for total RNA and protein extraction.

### Microarray Analysis

Total RNA from HESCs treated with steroids for 6h was isolated using miRNeasy Mini Kit and RNeasy MinElute Cleanup Kit according to the manufacturer’s instructions (Qiagen, Valencia, CA). The quality of isolated RNAs was confirmed using an Agilent 2100 Bioanalyzer. Extracted total RNAs were sent to the Keck Biotechnology Resource Laboratory at Yale University (New Haven, CT, USA) for microarray analysis using Illumina Human HT–12 v4 Expression BeadChip (Illumina Inc., San Diego, CA). Microarray analysis was limited to specimens whose RNA Integrity Number (RIN) value exceeded 8. Sample labeling and hybridization was performed per manufacturer’s instructions. Raw data without normalization were analyzed by GeneSpring GX12.5 software (Agilent Technologies-Silicon Genetics, Redwood City, CA). Gene readouts were normalized to the 75^th^ percentile of the distribution of all measurements in each chip. Normalization for each gene across chips was performed using the median value of each gene throughout different chips in the same experimental condition. Normalized data were first filtered to eliminate the genes, which were absent in all experimental conditions and replicates. The resulting data were filtered on volcano plot with moderated *t*-test without multiple testing corrections. Genes with a fold change of > 1.25 and a *p*-value of < 0.05 were considered differentially expressed. Molecular functions and biological networks related to differentially expressed genes were explored using Ingenuity Pathway Analysis (IPA) software (Ingenuity Systems, Redwood City, CA) according to manufacturer’s instructions. The data discussed in this publication have been deposited in NCBI's Gene Expression Omnibus and are accessible through GEO Series accession number GSE72040 (http://www.ncbi.nlm.nih.gov/geo/query/acc.cgi?acc=GSE72040).

### Reverse Transcription and Quantitative Real Time (q)-PCR Analysis

Total RNA from cultured HESCs was isolated using miRNeasy mini kit and RNeasy MinElute cleanup kit according to the manufacturer’s instructions (Qiagen). Reverse transcription was performed using RETROscript kit (Ambion, Austin, TX) in two steps: first, 2 μg of total RNA from each sample was incubated with random decamer primers at 85°C for 3 min to eliminate any secondary structures, then a reverse transcription reaction was carried out at 42°C for 1 h consisting of 2 μl of 10X reverse transcription buffer, 4 μl of 1.25 mM dNTP mix, 1 μl of RNase inhibitor (10 units/μl) and 1 μl of MMLV-reverse transcriptase enzyme (100 units/μl). Subsequently, inactivation of the reverse transcriptase enzyme at 92°C for 10 min was performed.

The expression of FKBP51, insulin-like growth factor binding protein 2 (IGFBP2), four jointed box 1 (FJX1), thrombin receptor like–2 (F2RL2, aka proteinase-activated receptor–3; PAR3), ADAM metallopeptidase with thrombospondin type 1 motif, 1 (ADAMTS1), IL-1β, and β-actin was determined by q-PCR using TaqMan Gene Expression Assay according to the manufacturer's protocol. In brief, 10 μL of TaqMan 2× Universal PCR Master Mix was combined with 8.5 μL of nuclease-free water, 1 μL of 20× TaqMan primer/probe mix, and 0.5 μL of cDNA in the PCR tube. Amplification used 40 cycles of PCR in Applied Biosystems 7500 Real-Time PCR Detection System (Applied Biosystems, Foster City, CA), with the following program: initial denaturation at 95°C for 10 minutes, followed by 40 cycles at 95°C for 15 seconds and 60°C for 60 seconds. All samples were run in triplicate and the average used for each sample. A standard curve for each set of primers was first used to determine the linear dynamic range of each reaction and the PCR efficiency. Expression of the target mRNAs was normalized to β-actin levels and the 2^−ΔΔCt^ (cycle threshold) method was used to calculate relative expression levels. Results are reported as fold change in gene expression levels among the different groups. The following TaqMan gene expression assays were used: FKBP51 (ID# Hs01561006_m1); IGFBP2; (ID# Hs01040719_m1); FJX1 (ID# Hs00534909_m1); F2RL2 (ID#Hs00765740); ADAMTS1 (ID# Hs00199608_m1); IL-1β (ID#Hs1555410_m1); and β-actin (ID# Hs99999903_m1) (Applied Biosystems).

### FKBP51 overexpression

To over-expression of FKBP51 in HESCs, expression vector containing the full-length FKBP51 gene open reading frame (pCMV6-FKBP51) and empty vector (pCMV6) were purchased from Origene Inc. (Rockville, MD). Transient transfection were performed in HESC (approximately 70% confluence) using Lipofectamine LTX reagent (Invitrogen, Carlsbad, CA) in Opti-MEM I serum reduced medium (Invitrogen) according to manufacturer’s instructions. Following 44 h of transfection, HESC were incubated with E_2_ alone or E_2_+MPA±thrombin (1U/ml, American Diagnostic, Greenwich, CT) for 4h then washed with ice-cold PBS twice and stored in -80°C for subsequent experiments. Transfection efficiency for over-expression of FKBP51 was confirmed by q-PCR and immunoblotting analyses.

### Immunoblot Analysis

To evaluate FKBP51 protein levels, cell lysates from HESCs cultured for 24h with E_2_ or E_2_+P4 or E_2_+MPA or E_2_+ETO were diluted in 2X sample buffer (Bio-Rad, Hercules, CA) and then boiled for 5 minutes. The cell lysates were subjected to reducing SDS-PAGE on a 10% Tris-HCl gel (Bio-Rad), with subsequent electroblotting transfer onto a 0.45-μm nitrocellulose membrane (Bio-Rad). After transfer, the membranes were blocked overnight in Tris-Buffered Saline (TBS) with 10% non-fat dry milk and then incubated with goat anti-FKBP51 polyclonal antibody at 1/1000 dilution (R&D Systems) or with rabbit anti-total (T) and phosphorylated (P)-AKT, or with rabbit anti-T and P-ERK1/2 MAPK antibodies (Cell Signaling) at 1/1000 dilution for primary antibody labeling. Membranes were rinsed in TBS-T (TBS with 0.1% Tween 20) subsequently incubated with peroxidase-conjugated anti-goat IgG at 1/5000 dilution (Vector Labs) and signals were developed using a chemiluminescence kit (Amersham; GE Healthcare, Piscataway, NJ). The membranes was sequentially stripped and re-probed with peroxidase-conjugated anti-β-actin rabbit monoclonal antibody at 1/1000 dilution (Cell Signaling Technology).

### Guinea Pig Studies

Endometrial samples were obtained from oophorectomized (OVX) GPs treated in parallel with placebo or E_2_ or MPA or E_2_+MPA under the approval of Yale Institutional Animal Care and Use Committee (IACUC#2006–11002). Twelve nulliparous GPs, aged 2–6 months, were subjected to bilateral oophorectomy. Three GPs for each treatment group were given subcutaneous 50 mg MPA, cholesterol-based 21 day time-release (2.4 mg/day) pellets or 5 mg E_2_ cholesterol based 21 day time-release (240 μg/day) pellets or E_2_+MPA or placebo pellets (Innovative Research of America; Sarasota, FL). After three weeks, hysterectomy was performed and the right uterine horn was formalin-fixed for immunohistochemical studies.

### Human Endometrial Tissues

Paraffin sections were derived from previously banked paired endometrial tissues obtained prior to DMPA injection (pre-DMPA) in the secretory phase and 80 to 90 days after initial DMPA injection (post-DMPA). Endometrial aspiration biopsy was used to obtain samples from woman with regular menstrual cycles during the secretory phase (n = 6), while hysteroscopic biopsy was used to obtain endometrial specimens from women following DPMA injection after receiving written informed consent at New York University under Institutional Review Board approval (#H6023) and IRB approval of University of South Florida (#Pro00019480). All samples used in our study were de-identified when they were obtained.

### Immunohistochemistry

Paraffin sections of human and GP endometria were deparaffinized in xylene and rehydrated through a descending ethanol series. Antigen retrieval involved boiling the slides in citrate buffer (pH:6.0) followed by endogenous peroxidase quenching with 3% hydrogen peroxide and blocking non-specific antibody binding with 5% normal horse serum (Vector Labs). The sections were incubated overnight at 4°C with goat anti-FKBP51 polyclonal IgG at 1/200 dilution (R&D Systems). After several rinses in TBS-T, slides were incubated with biotinylated horse anti-goat IgG at 1/5000 dilution (Vector Labs) for 30 min, rinsed in TBS-T x3 and then incubated with a streptavidin-peroxidase complex (Elite ABC kit; Vector Labs). Diaminobenzidine tetrahydrochloride dehydrate (DAB; Vector Labs) was used as a chromogen (brown stain) to visualize immunoreactivity. Hematoxylin was used for background staining. Non-immune goat IgG was used at the same concentration as the primary antibody as a negative control.

The intensity of FKBP51 immunostaining was semi-quantitatively evaluated by HSCORE analysis and categorized into the following scores: 0 (no staining), 1+ (weak, but detectable, staining), 2+ (moderate staining), and 3+ (intense staining). An HSCORE value was derived for each specimen by calculating the sum of the percentage of cells stating in each category multiplied by its respective intensity score using the formula HSCORE = ∑_i_ i*Pi, where i represents the intensity category score, and Pi is the corresponding percentage of cells [24]. For each slide, five different fields were evaluated microscopically at 200Χ magnification. HSCORE evaluation was performed independently by two investigators blinded to the source of the samples; the average score of both was then used.

### Database search of Transcription factor binding sites

The MatInspector and GeneCards program (http://www.genomatix.de and http://www.genecards.org) were used to identify GR binding sites.

## Statistical analysis

Immunostaining HSCOREs of FKBP51 in GP endometria, immunoblotting and q-PCR results were each normally distributed as determined by Kolmogorov-Smirnov test and each data set analyzed by One-way ANOVA followed by testing post-hoc Holm-Sidak method. *p<0*.*05* is accepted as statistically significant. Immunostaining HSCOREs for human endometrial tissues from pre- and post-DMPA administration were compared using a *t*-test. Statistical calculations used SigmaStat version 3.0 software (Systat Software, San Jose, CA).

## Results

### MPA or ETO modulated gene expression in HESCs

The results of whole genome microarray analysis of cultured HESCs displayed in Table 1 indicate that compared with treatment with E_2_, E_2_+MPA or E_2_+ETO significantly altered transcription of 378 and 538 genes, respectively, whereas E_2_+P_4_ modified transcription of only 171 genes. Specifically, among: 1) 378 differentially regulated genes by E_2_+MPA, 222 genes (59%) were upregulated and 156 (41%) were downregulated; 2) 538 differentially regulated genes by E_2_+ETO, 263 (49%) were upregulated and 275 (51%) were downregulated; 3) 171 differentially regulated genes by E_2_+P_4_, 150 (88%) were upregulated and 21 (12%) were downregulated (Table 1). The 40 most highly regulated genes in HESCs in response to E_2_+MPA or E_2_+ETO or E_2_+P_4_ were given in Tables 2–4.

**Table 1:** Comparison of number of differentially regulated genes in cultured HESCs treated by E_2_+MPA, E_2_+ETO and E_2_+P_4_ *vs*. E_2_ according to whole genome microarray analysis using Illumina HumanHT–12 v4 expression BeadChip kit analysis (n = 3/group).

	Total number of genes altered	Number of genes upregulated	Number of genes downregulated
**E**_**2**_ **+MPA (n = 3)**	378	222 (59%)	156 (41%)
**E**_**2**_ **+ETO (n = 3)**	538	263 (49%)	275 (51%)
**E**_**2**_ **+P4 (n = 3)**	171	150 (88%)	21 (12%)

**Table 2:** 40 most highly differentially regulated genes in HESCs treated by E_2_+MPA *vs*. E_2_ alone according to whole genome microarray analysis using Illumina HumanHT–12 v4 expression BeadChip kit. All gene symbols were abbreviated according to GENEBANK standard nomenclature.

Symbol	Fold Change	Location	Function
PARM1	3.897	Extracellular Space	Other
ADAMTS1	2.995	Extracellular Space	Peptidase
CLEC3B	2.963	Extracellular Space	Other
IGFBP2	2.832	Extracellular Space	Other
ADH1A	2.724	Cytoplasm	Enzyme
CRISPLD2	2.621	Cytoplasm	Other
CILP	2.553	Extracellular Space	Phosphatase
AOX1	2.550	Cytoplasm	Enzyme
APOD	2.541	Extracellular Space	Transporter
ZBTB16	2.536	Nucleus	transcription regulator
CFD	2.465	Extracellular Space	Peptidase
MAOA	2.415	Cytoplasm	Enzyme
MUM1L1	2.301	Cytoplasm	Other
MEDAG	2.139	Cytoplasm	Other
**FKBP5 (FKBP51)**	2.133	Nucleus	Enzyme
CHST7	2.109	Cytoplasm	Enzyme
TSC22D3	2.103	Nucleus	transcription regulator
IMPA2	2.068	Cytoplasm	Phosphatase
ITPR1	2.057	Cytoplasm	ion channel
PPAP2B	2.015	Plasma Membrane	Phosphatase
FJX1	-3.341	Extracellular Space	Other
MMP11	-3.047	Extracellular Space	Peptidase
IL17RB	-2.651	Plasma Membrane	transmembrane receptor
COL8A1	-2.137	Extracellular Space	Other
TENM4	-2.104	Other	Other
TFRC	-2.091	Plasma Membrane	Transporter
EGR2	-2.055	Nucleus	transcription regulator
MYLIP	-1.951	Cytoplasm	Enzyme
SFRP4	-1.879	Plasma Membrane	transmembrane receptor
IRS1	-1.797	Cytoplasm	Enzyme
AFAP1L2	-1.784	Cytoplasm	Other
PAMR1	-1.765	Extracellular Space	Peptidase
F2RL1	-1.751	Plasma Membrane	G-protein coupled receptor
PGR	-1.744	Nucleus	ligand-dependent nuclear receptor
BMP6	-1.726	Extracellular Space	growth factor
STC2	-1.724	Extracellular Space	Other
F2RL2	-1.701	Plasma Membrane	G-protein coupled receptor
ETV5	-1.68	Nucleus	transcription regulator
KAL1	-1.677	Extracellular Space	Other
CDKN2B	-1.663	Nucleus	transcription regulator

**Table 3:** 40 most highly differentially regulated genes in HESCs treated by E_2_+ETO *vs*. E_2_ alone according to whole genome microarray analysis using Illumina HumanHT–12 v4 expression BeadChip kit. All gene symbols were abbreviated according to GENEBANK standard nomenclature.

Symbol	Fold Change	Location	Function
PARM1	4.546	Extracellular Space	other
ADAMTS1	3.293	Extracellular Space	peptidase
IGFBP2	3.117	Extracellular Space	other
APOD	3.068	Extracellular Space	transporter
ZBTB16	2.972	Nucleus	transcription regulator
ALDH1A3	2.820	Cytoplasm	enzyme
ADH1A	2.717	Cytoplasm	enzyme
CLEC3B	2.705	Extracellular Space	other
MUM1L1	2.648	Cytoplasm	other
MAOA	2.592	Cytoplasm	enzyme
CRISPLD2	2.588	Cytoplasm	other
CHST7	2.580	Cytoplasm	enzyme
CILP	2.442	Extracellular Space	phosphatase
**FKBP5(FKBP51)**	2.357	Nucleus	enzyme
AOX1	2.353	Cytoplasm	enzyme
IMPA2	2.348	Cytoplasm	phosphatase
RGS2	2.335	Nucleus	other
PPAP2B	2.186	Plasma Membrane	phosphatase
MEDAG	2.179	Cytoplasm	other
CFD	2.137	Extracellular Space	peptidase
FJX1	-3.325	Extracellular Space	other
COL8A1	-2.569	Extracellular Space	other
IL17RB	-2.263	Plasma Membrane	transmembrane receptor
MMP11	-2.239	Extracellular Space	peptidase
TFRC	-2.137	Plasma Membrane	transporter
TENM4	-2.100	Other	other
GREM1	-2.019	Extracellular Space	other
MYLIP	-1.984	Cytoplasm	enzyme
IRS1	-1.966	Cytoplasm	enzyme
ETV5	-1.946	Nucleus	transcription regulator
EGR2	-1.939	Nucleus	transcription regulator
TNFAIP6	-1.924	Extracellular Space	other
BMP6	-1.890	Extracellular Space	growth factor
F2R	-1.861	Plasma Membrane	G-protein coupled receptor
SFRP4	-1.849	Plasma Membrane	transmembrane receptor
STC2	-1.848	Extracellular Space	other
F2RL1	-1.837	Plasma Membrane	G-protein coupled receptor
CDKN2B	-1.803	Nucleus	transcription regulator
PGR	-1.775	Nucleus	ligand-dependent nuclear receptor
CADM1	-1.758	Plasma Membrane	other

**Table 4:** 40 most highly differentially regulated genes in HESCs treated by E_2_+P_4_ *vs*. E_2_ alone according to whole genome microarray analysis using Illumina HumanHT–12 v4 expression BeadChip kit. All gene symbols were abbreviated according to GENEBANK standard nomenclature.

Symbol	Fold Change	Location	Function
CLEC3B	2.217	Extracellular	Other
ADH1A	1.973	Cytoplasm	enzyme
SPARCL1	1.970	Extracellular	other
PDGFD	1.835	Extracellular	growth factor
IGFBP2	1.819	Extracellular	other
ADAMTS1	1.714	Extracellular	peptidase
CILP	1.701	Extracellular	phosphatase
CRISPLD2	1.607	Cytoplasm	other
AOX1	1.528	Cytoplasm	enzyme
LMO3	1.517	Other	other
TIMP3	1.516	Extracellular	other
BCHE	1.513	Plasma Membrane	enzyme
OMD	1.498	Other	other
SULF2	1.477	Plasma Membrane	enzyme
GAS1	1.466	Plasma Membrane	other
LAMA2	1.461	Extracellular	other
RPL14	1.435	Other	other
MASP1	1.431	Extracellular	peptidase
TMEM45A	1.430	Plasma Membrane	other
FBLN5	1.426	Extracellular	other
SERPING1	1.421	Extracellular	other
ANTXR1	1.418	Plasma Membrane	transmembrane receptor
IL17RB	-1.926	Plasma Membrane	transmembrane receptor
MMP11	-1.794	Extracellular	peptidase
FJX1	-1.682	Extracellular	other
COL8A1	-1.664	Extracellular	other
VGF	-1.404	Extracellular	growth factor
SDC1	-1.399	Plasma Membrane	enzyme
TENM4	-1.383	Other	other
SFRP4	-1.383	Plasma Membrane	transmembrane receptor
AFAP1L2	-1.374	Cytoplasm	other
DPYSL4	-1.358	Cytoplasm	enzyme
HSPE1	-1.354	Cytoplasm	enzyme
TFRC	-1.339	Plasma Membrane	transporter
KCNIP4	-1.292	Plasma Membrane	ion channel
MYLIP	-1.291	Cytoplasm	enzyme
GADD45G	-1.262	Nucleus	other
DBNDD1	-1.259	Other	other
RFX7	-1.257	Other	other
RALGDS	-1.251	Cytoplasm	other

**Fig 1:**
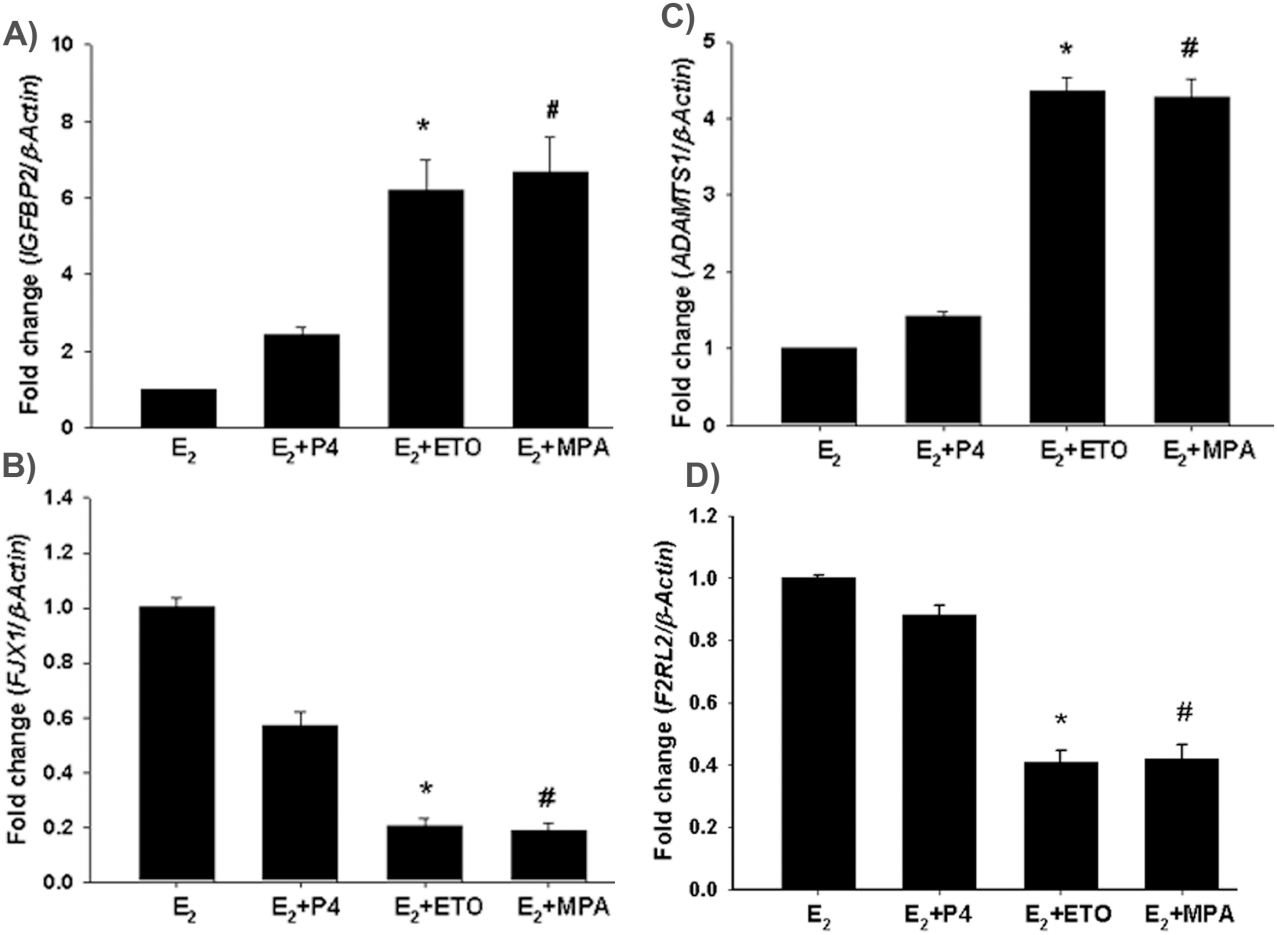
Conformational evaluation of genes commonly regulated by all three progestins. q-PCR for (A) *IGFBP2*, (B) *FJX1*, (C) *ADAMTS1* and (D) *F2RL2* (**D**) in HESCs. HESC treated with estradiol (E_2_, 10^−8^M) ± progesterone (P_4_, 10^−7^ M) or etonogestrel (ETO, 10^−7^ M) or medroxyprogesterone acetate (MPA, 10^−7^ M) for 6h. Bars represent mean ± SEM (n = 3). * *p<0*.*001* in E_2_+ETO *vs*. E_2_; # *p<0*.*001* in E_2_+MPA *vs*. E_2_.

Several of the highly regulated genes (Tables 2–4) identified by microarray analysis responded to either MPA or ETO, but not to P_4_. This observation suggested that modulation of these genes by MPA and ETO may reflect a glucocorticoid action. This observation was supported by use of the web-based programs i.e. MatInspector and GeneCards (http://www.genomatix.de and http://www.genecards.org) which identified the presence of GREs in the promoter of the IGFBP2, ADAMTS1, F2RL2 and FKBP51 genes, but not in the FJX1 gene promoter (S1 Fig). Thus, these MPA and ETO specific regulated genes obtained from microarray analysis were confirmed by performing q-PCR on five genes (IGFBP2, FJX1, F2RL2, ADAMTS1, and FKBP51) found to be differentially regulated in HESCs following treatment with E_2_+MPA or E_2_+ETO (Fig 1A–1D). In parallel with microarray results, IGFBP2 and ADAMTS1 mRNA levels are significantly upregulated by E_2_+ETO or E_2_+MPA (Fig 1A and 1C), whereas FJX1 and F2RL2 mRNA levels are significantly downregulated by E_2_+MPA and E_2_+ETO (Fig 1B and 1D). By comparison, none of the modest responses elicited by P4 for each of the endpoints attained statistical significance (Fig 1A–1D).

**Fig 2:**
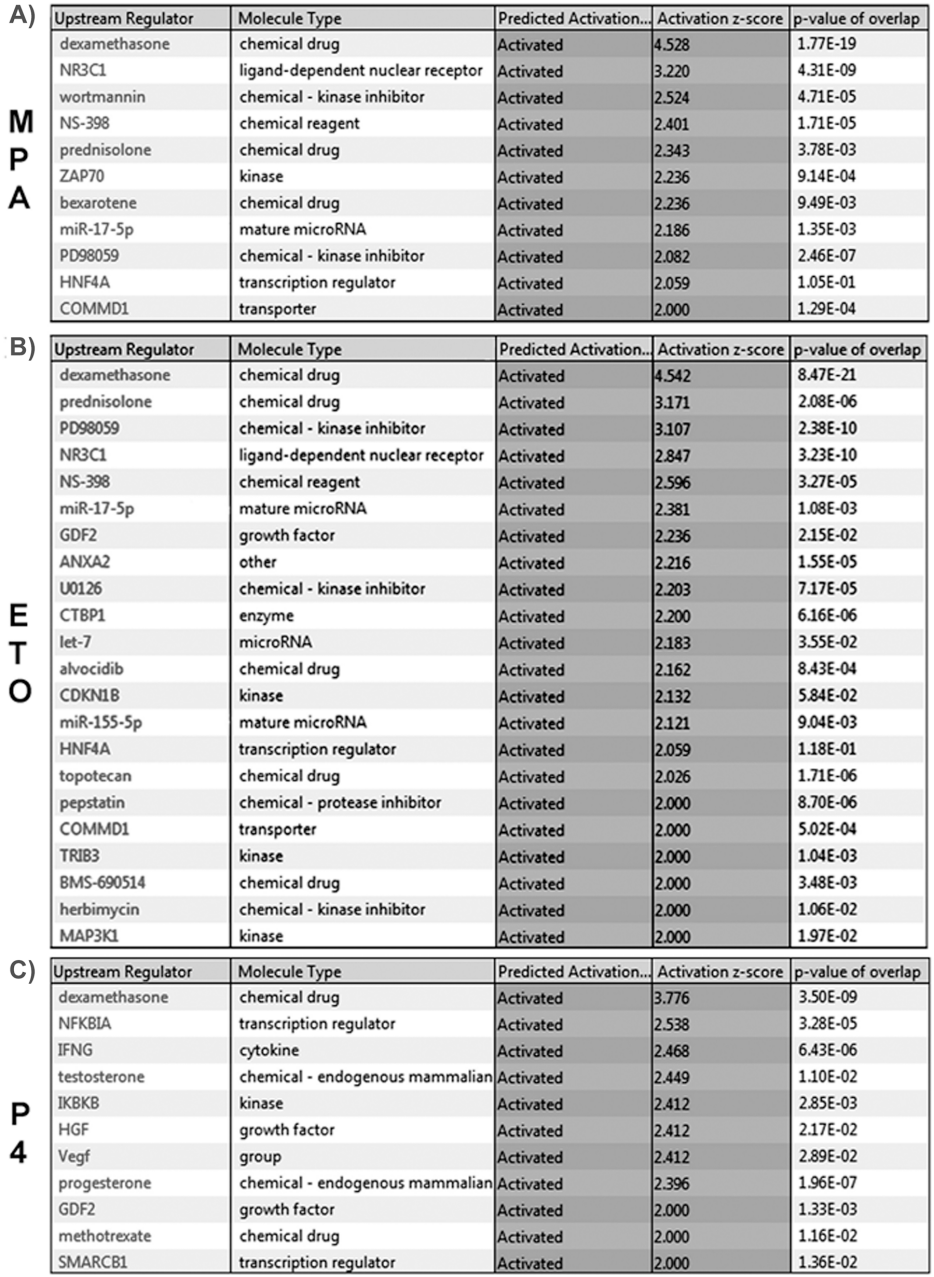
Common and unique upstream regulator of differentially regulated genes by MPA, ETO and P_4_. Ingenuity Pathway Analyzer (IPA) software identifies activation of ligand-dependent glucocorticoid receptor (GR,NR3C1) by (A) MPA (*Z*-score 3.22; overlap *p* value 4.31 E^−9^) and (B) ETO (*Z*-score 2.85; overlap *p* value 3.22 E^−10^), but not (C) P_4_ (*Z* score = 0 and overlap *p* value = 0.03). All gene symbols were abbreviated according to GENEBANK standard nomenclature. (n = 3/group).

Evaluation of the microarray results using IPA revealed associations between differentially regulated gene clusters and activation of several common and unique upstream regulators in HESCs in response to either E_2_+MPA or E_2_+ETO or E_2_+P_4_ treatment (see *Z*-value and overlap *p*-value for each progestogen; Fig 2). Among the signaling pathways revealed by IPA, inspection of Fig 2 indicates that genes altered by each of the progestins are associated with activation of dexamethasone mediated signaling, whereas genes altered by treatment with E_2_+ETO or E_2_+MPA (Fig 2A and 2B), but not E_2_+P_4_ (Fig 2C), are associated with ligand-dependent activation of GR (NR3C1, see S2 Fig), PD98059 (ERK1/2 MAPK inhibitor) and miR-17-5p (a mature cytoplasmic micro-RNA), hepatocyte nuclear factor 4, alpha (HNF4A) (Fig 2A and 2B). In contrast with the modulation of common genes by either E_2_+ETO or E_2_+MPA, genes regulated by E_2_+P_4_ treatment are uniquely associated with upstream activation of nuclear factor of kappa light polypeptide gene enhancer in B-cells inhibitor alpha, aka IĸBα (NFKBIA), IFNG (interferon γ), P4, VEGF (Fig 2C).

**Fig 3:**
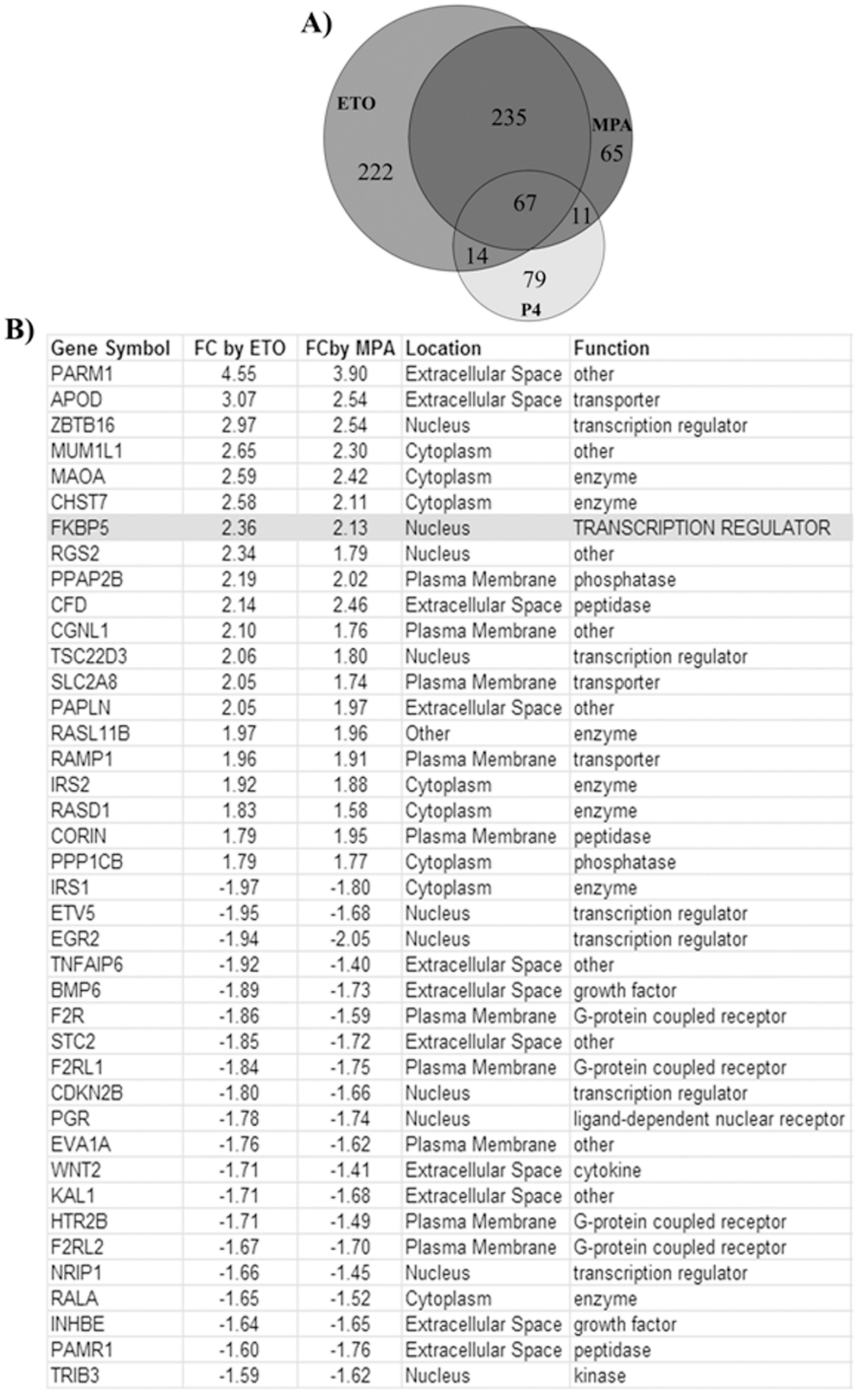
MPA, ETO and P_4_ mediated differentially regulated genes in HESCs. (A) Venn diagram analysis of genes regulated individually or in common by MPA or ETO or P_4_ (B) 40 most highly regulated genes by ETO and MPA, but not P_4_ according to whole genome microarray analysis using Illumina HumanHT–12 v4 expression BeadChip kit (n = 3/group). All gene symbols were abbreviated according to GENEBANK standard nomenclature.

Further analysis of the microarray data displayed by Venn diagram indicates that 65 genes are regulated solely by E_2_+MPA, and 222 genes are regulated solely by E_2_+ETO whereas 79 genes are regulated solely by E_2_+P_4_ (Fig 3A). A group of 67 genes are regulated in common by all three progestins. Strikingly, transcriptional levels of 235 genes are regulated in common by E_2_+ETO and E_2_+MPA, but not by E_2_+P_4_ (Fig 3A). Among these, the 40 most highly regulated genes by ETO and MPA are listed in Fig 3B. IPA of the microarray gene expression data revealed the activation of GR (NR3C1) as one of upstream regulators of several of these 235 genes regulated by ETO and MPA (S2 Fig).

### MPA and ETO induce FKBP51 mRNA and protein expression in cultured HESCs

**Fig 4:**
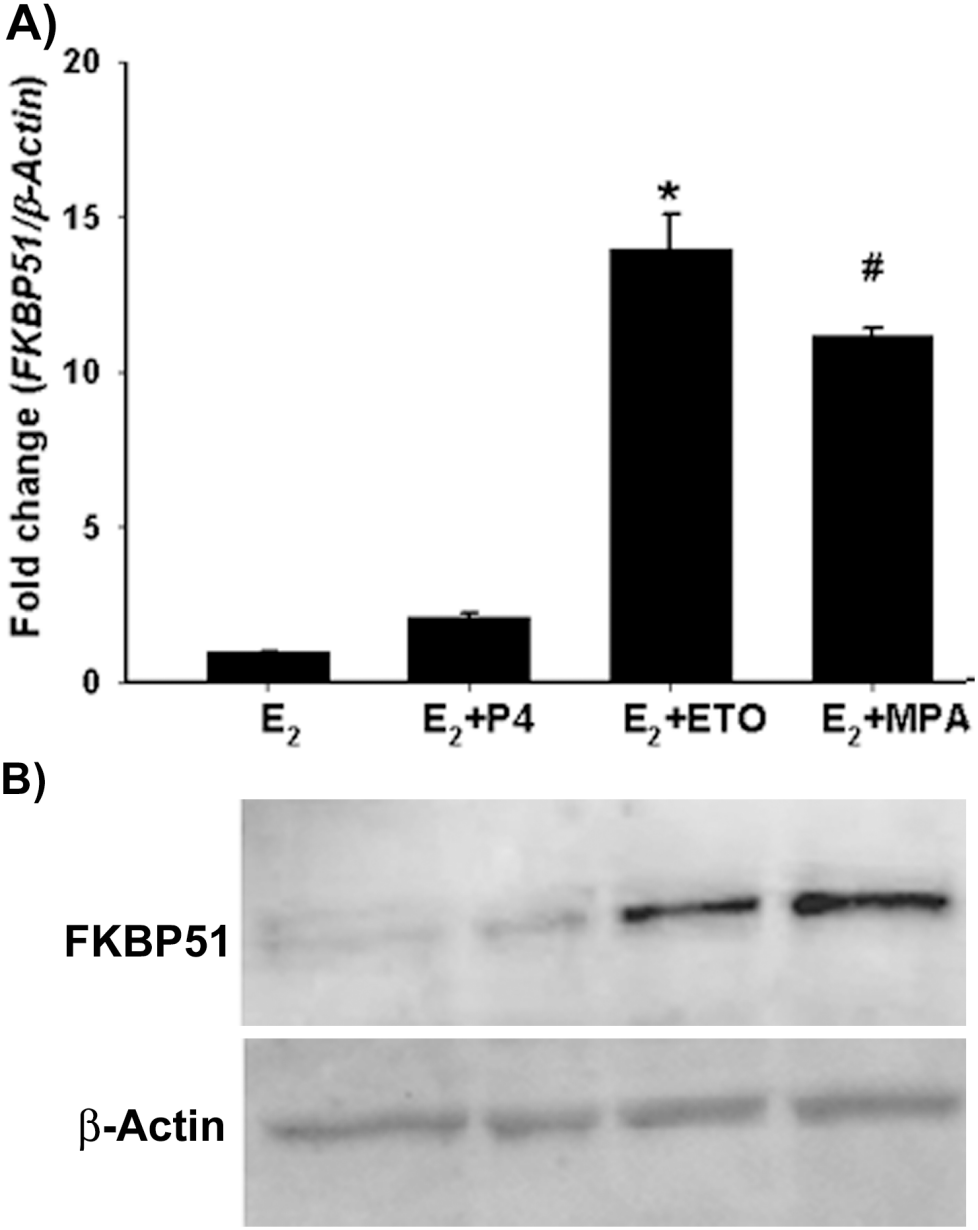
Increased FKBP51 mRNA and protein levels by MPA or ETO but not P4. (A) q-PCR and (B) immunoblot analysis of FKBP51 levels in HESCs treated with estradiol (E_2_, 10^−8^M) ± progesterone (P_4_, 10^−7^ M) or etonogestrel (ETO, 10^−7^ M) or medroxyprogesterone acetate (MPA, 10^−7^ M) for 6h or 24 h, respectively. Bars represent mean ± SEM (n = 3). * *p<0*.*001* in E_2_+ETO *vs*. E_2_; # *p<0*.*001* in E_2_+MPA *vs*. E_2_.

FKBP51 represses GR and PR transcriptional activity in several cell types [18, 20, 25] and is highlighted in bold among the genes specifically altered by either E_2_+ETO or E_2_+MPA treatment (Fig 3B). Analysis by q-PCR confirmed that incubation of HESCs with E_2_+ETO or E_2_+MPA, but not E_2_+P_4_, significantly induces steady state FKBP51 mRNA levels *vs*. E_2_ alone (Fig 4A) and, immunoblot analysis revealed that compared with E_2_ alone, FKBP51 protein levels are increased significantly in HESCs in response to E_2_+ETO or E_2_+MPA, but not to E_2_+P_4_ (Fig 4B). Unlike FKBP51, microarray analysis revealed no change in FKBP52 mRNA expression in response to all three progestins and immunoblotting confirmed that none of the three progestins affected FKBP52 protein expression (not shown).

### Endometrial FKBP51 expression in women receiving DMPA treatment

**Fig 5:**
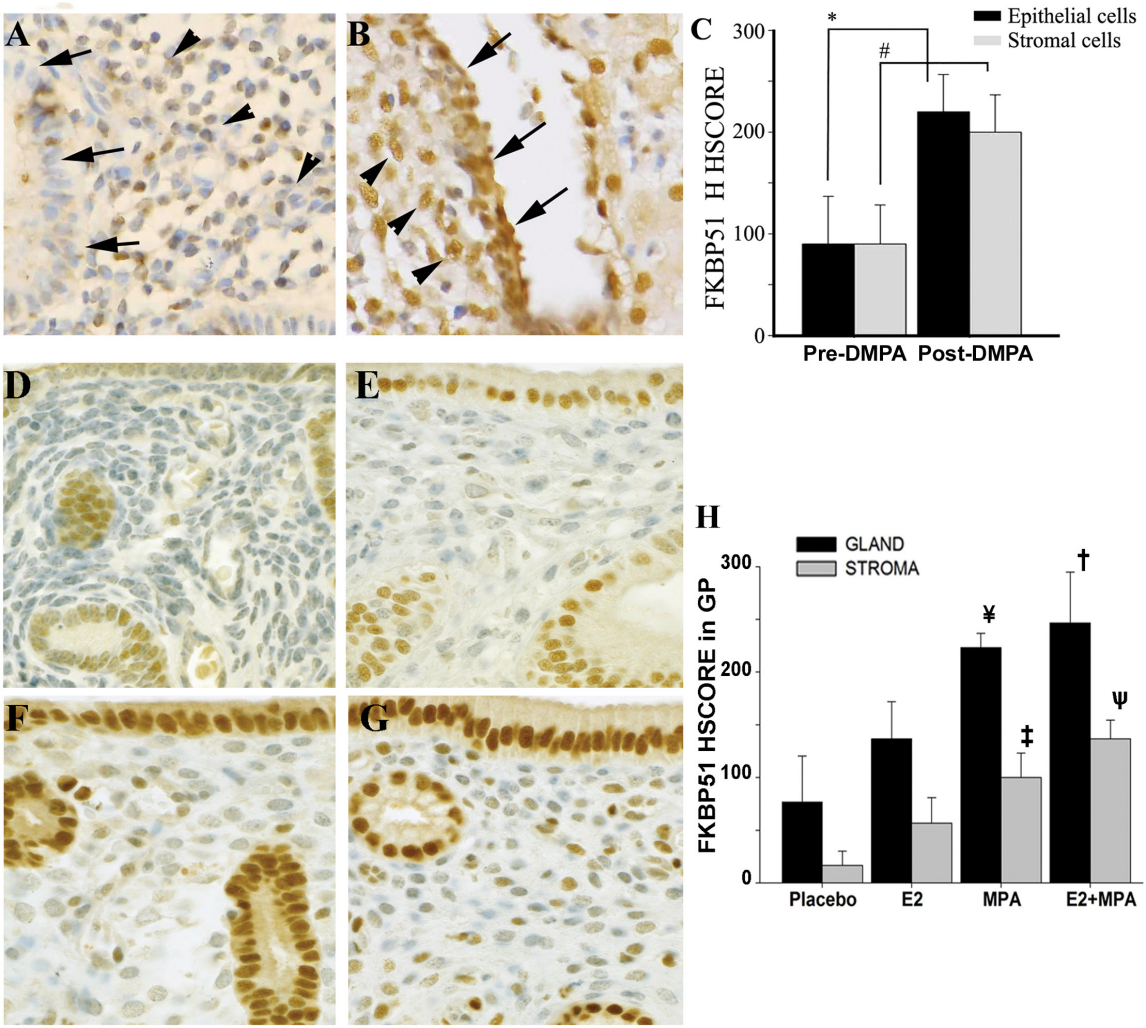
Increased FKBP51 immunoreactivity in women using DMPA and in endometria of OVX-GPs treated by MPA. (A_C) FKBP51 expression in paired endometrium of women pre- and post- DMPA administration. Immunoreactive FKBP51 in endometrial stromal (arrowheads) and glandular (arrow) cells pre- (A) and post- (B) DMPA use. HSCORE analysis of FKBP51 expression (C) in stromal and glandular cells. Bars represent mean ± SEM (n = 6). * *p< 0*.*01* and ^#^ *p<0*.*01*. Pre-DMPA: before DMPA use; Post-DMPA: 3 months DMPA use. (D-H) FKBP51 expression in endometria of OVX-GPs treated by MPA. Immunoreactivity for FKBP51 (brown) in stromal (arrowhead) and epithelial (arrows) cells of endometria of OVX-GPs treated with vehicle (D) or E_2_ (E) or MPA (F) or E_2_+MPA (G) for 21 days. HSCORE analysis of FKBP51 immunoreactivity (H) in stromal and glandular cells. Bars represent mean ± SEM (n = 3 per treatment group). **†** *p<0*.*001* in E_2_+MPA *vs*. E_2_ or placebo (Pla); **ѱ** *p<0*.*001* in E_2_+MPA *vs*. MPA or E_2_ or Pla; **¥** in *p<0*.*05* in MPA *vs*. E_2_ or Pla; **‡** *p<0*.*05* in MPA *vs*. E_*2*_ and Pla. Original Magnification: A-G x40.

Immunohistochemical analysis was performed on paired endometrial sections obtained from women pre- and post-DMPA administration. Both endometrial stromal and glandular epithelial cells express weak to moderate FKBP51 immunoreactivity in pre-DMPA specimens (Fig 5A). Post-DMPA specimens displayed a significant increase in FKBP51 immunoreactivity in endometrial stromal cells (HSCORE mean ± SEM; 200 ± 18.3 *vs*. 100 ± 14.1; *p<0*.*009* respectively) and endometrial glandular epithelial cells (220 ± 18.3 *vs*. 92.5 ± 22.1; *p<0*.*006*, respectively) compared with pre-DMPA administration (Fig 5B and 5C).

### MPA induces FKBP51 expression in the endometrium of OVX-GPs

FKBP51 immunoreactivity in endometrial sections obtained from OVX-GPs treated with placebo or E_2_ or MPA or E_2_+MPA is displayed in Fig 5D–5H. In endometrial stromal cells, FKBP51 expression is significantly increased by administration of MPA (HSCORE mean ± SEM; 100.0 ± 11.5) or E_2_+MPA (136.7 ± 8.8) compared to placebo (16.7 ± 6.7) or E_2_ alone (56.7 ± 12.02; *p<0*.*001*, Fig 5D–5G). Similar increases in FKBP51 expression were also observed in endometrial glandular epithelial cells of GPs treated with either MPA (223.3 ± 6.7) or E_2_+MPA (246.7 ± 24.1) compared to placebo (76.7 ± 21.9) or E_2_ alone (136.7 ± 7.6; *p<0*.*001*, Fig 5D–5G). Consistent with E_2_ mediated up-regulation of PR expression [26], HSCORE analysis (Fig 5H) indicates that E_2_+MPA significantly enhanced FKBP51 expression in endometrial stromal cells *vs*. MPA alone (*p<0*.*05*). No statistically significant differences between placebo and E_2_ alone were detected in either endometrial stromal or glandular epithelial cells (Fig 5H).

### Overexpression of FKBP51 reverses MPA-mediated inhibition of endogenous IL-1β expression in HESCs

**Fig 6:**
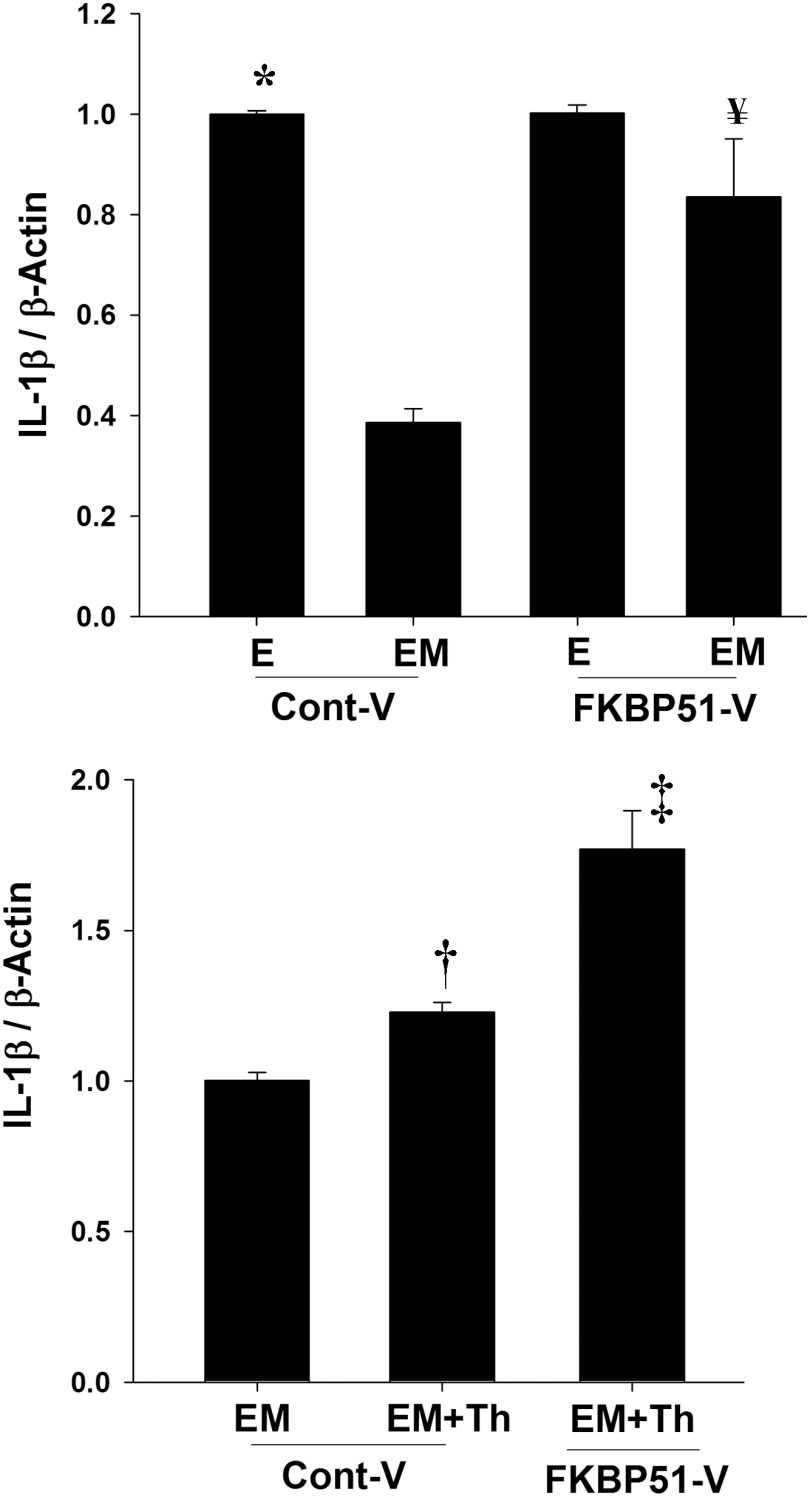
FKBP51 overexpression reverses the anti-inflammatory action of MPA in HESCs. (A) Basal *IL-1β* mRNA levels in 10^−8^ M estradiol (E) *vs*. E+ 10^−7^ M MPA (EM) treated HESCs at 44 hrs post-transfection with either a control (Cont-V) or FKBP51 vector (FKBP51-V). Bars represent mean ± SEM (n = 4 with 3 replicates per treatment group). (B) In parallel incubations, thrombin- (Th-) induced *IL-1β* mRNA levels in E_2_+MPA treated HESCs at 44 hrs post-transfection with control or FKBP51 vector. Bars represent mean ± SEM (n = 2 and 3 replicates per treatment group). E: E_2_ and EM: E_2_+MPA, * *p<0*.*05 vs*. EM treated in Cont-V transfected HESCs; **¥** *p<0*.*05 vs*. EM treatment in Cont-V transfected HESCs; **†** *p<0*.*05 vs*. EM; **‡** *p<0*.*05 vs*. EM+Th.

Our previous studies showed that LAPCs increase TF expression in decidualized HESCs, which generates excess thrombin [11, 12]. In cultured HESCs, thrombin enhances expression of VEGF, IL–8, and MMP–1 expression [13, 14, 23] and therefore, thrombin plays an essential role in LAPC-induced AUB by promoting a proteolytic and inflammatory milieu. To investigate the effect of FKBP51 in MPA-mediated inhibition of inflammation, IL-1β mRNA levels were measured in HESCs transfected with FKBP51 vector for overexpression. The FKBP51 expression was confirmed by q-PCR and immunoblotting in cultured HESCs (data not shown) after transfection. Compared with E_2_ treatment, E_2_+MPA treatment significantly inhibited *IL-1β* mRNA levels in HESC transfected with a control vector, whereas MPA failed to inhibit *IL-1β* mRNA levels in HESCs overexpressing FKBP51 (Fig 6A). Furthermore, thrombin treatment of HESCs that overexpressed FKBP51 exacerbates thrombin-induced *IL-1β* mRNA expression in the presence of MPA (Fig 6B).

## Discussion

LAPC methods are the most effective reversible contraceptive method [21]. For example, in a large prospective cohort study in which over 75% of teenage participants received LAPCs there was a substantial reduction in mean annual rates of pregnancy, birth, and elective abortion compared with the overall population of sexually experienced U.S. teens [27]. However, AUB occurs in the majority of LAPC users and is a major reason for non-adherence, with three year discontinuation rates as high as 47% for implants and 27% for IUS users [28]. Moreover, unlike the predictable and controlled nature of menstrual bleeding, LAPC associated AUB is unpredictable and occurs sporadically [13]. The contraceptive action of LAPCs results from inhibition of ovulation, cervical thickening and/or endometrial thinning and explains the agents greater than 99% pregnancy preventing efficacy [29]. It is unclear how the various progestins employed in LAPC induce AUB. Seeking to shed light on this question, the current study identified several gene clusters and signaling pathways that are both uniquely and commonly modulated by each progestin. To our knowledge, this is the first study to compare changes in global gene expression profiles in HESCs in response to natural *vs*. synthetic progestins and consequently to identify common and unique alterations in gene expression triggered by each progestin.

During the follicular phase of the human menstrual cycle, circulating P4 is maintained at low levels that increase markedly following ovulation. This increased concentration does not induce uterine bleeding. Indeed it is the physiological withdrawal of circulating P4 that triggers menstrual bleeding [13, 30]. During human pregnancy circulating P4 levels continue to rise [31] unaccompanied by AUB. These physiological observations strongly suggest that AUB accompanying LAPC administration reflects molecular changes that are distinct from those induced by circulating P4. Consistent with this hypothesis, while our microarray results reveal that 65 genes are modulated in common by P4 and by the synthetic progestins, MPA and ETO, in cultured HESCs, these commonly modulated genes match only 17% and 12% of the total genes altered by MPA or ETO, respectively, and 38% of the genes modulated by P4 alone indicating that only a portion of the action of these LAPCs reflects native progesterone effects.

The current study identified a total of 235 genes unaffected during incubation of HESCs with P4, which corresponded to 62% and 44% of genes whose expression was altered by MPA and ETO, respectively. Further analysis by IPA software indicated ligand-dependent activation of GR (NR3C1) as one of the upstream regulators of these MPA or ETO-modulated 235 genes, suggesting the involvement of GR signaling in LAPC-induced AUB. Moreover, results from IPA predicted that activation of wortmannin, an AKT signal inhibitor, and PD89059, an ERK1/2 signal inhibitor, are other upstream signaling pathways associated with these genes. However, no changes in AKT or ERK1/2 phosphorylation levels were observed in HESCs treated with either MPA or ETO (S3 Fig), ruling out involvement of both signaling cascades in MPA and ETO-mediated modulation of these genes.

Prior studies demonstrated upregulation of FKBP51 mRNA and protein levels in response to ligand-dependent activation of both the GR and PR [17]. Microarray results obtained in the current study identified FKBP51 among genes that are upregulated following incubation of HESCs with either MPA or ETO, but not by P4. Confirmation of this selective elevation of FKBP51 mRNA was confirmed by q-PCR and extended to include the FKBP51 protein by immunoblotting. Lack of changes in FKBP52 expression in HESCs in response to MPA, ETO and P4 suggests that FKBP51 acts as a dominant co-chaperone in regulating PR or GR-mediated transcriptional activity in these cells. Reports that MPA displays mixed progestin/glucocorticoid effects [32] taken together with the absence of a response to P4 suggest that regulation of FKBP51 expression in HESCs by MPA as well as ETO reflects ligand-dependent activation of the GR, but not the PR. This observation is in contrast to information describing absence of glucocorticoid action by ETO. Alternatively, P4 is more susceptible to metabolism than synthetic progestins by cultured cells [33], which may reduce the effect of P4 on FKBP51 expression. Furthermore, compared with P4, MPA and ETO may exert a stronger PR mediated transcriptional activity on the FKBP51 gene. Supporting the latter statement, several genes presented in Tables 2–4 (such as ADAMTS1, ADH1A, CLEC3B) display higher fold increased transcriptional levels in response to MPA or ETO *vs*. P4. In several cell systems, elevated levels of FKPB51 suppresses transcriptional activity of both the GR and PR suggesting the existence of a negative-feedback loop that induces local functional P4 and GC withdrawal in target tissue [34]. The current observations of increased FKBP51 immunoreactivity in stromal and glandular cells in human endometrial tissues obtained from women from post-DMPA *versus* pre-DMPA use suggests that MPA administration results in local inhibition of GR and PR transcriptional activity. This may account for increase in inflammation observed in such endometria. In support of this hypothesis, several previous studies have shown a significant reduction in expression of tissue inhibitor of matrix metalloproteinases (TIMPs) in stromal, epithelial and endothelial cells, accompanied by focal elevation of MMP–1 and -3 expression and increased influx of MMP–9 positive neutrophils, CD3+ T cells, macrophages and uterine natural killer (uNK) cells in the endometria of DMPA users [35, 36]. Moreover, LAPCs increase epithelial neutrophil-activating peptide–78 (ENA–78) [37] and IL–8 in HESCs [15], and increase numbers of CD45 positive cells [35], which may be associated with functional loss of the anti-inflammatory properties of PR and GR activated by MPA or ETO.

**Fig 7:**
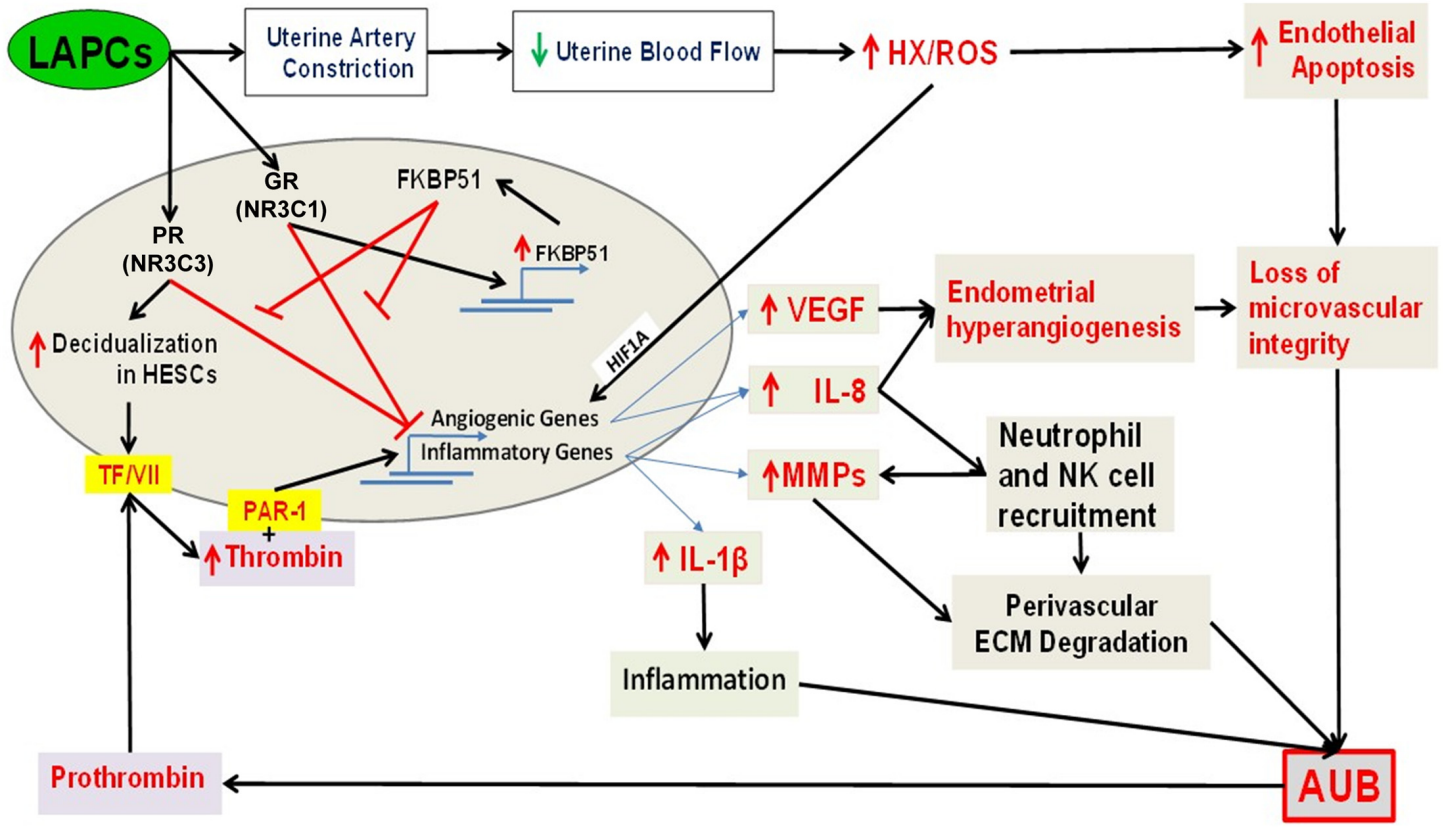
A schematic model for molecular interactions inducing AUB in women using LAPC. LAPCs reduce endometrial blood flow causing hypoxia (HX) and generating reactive oxygen species (ROS), to directly damage blood vessels and enhance HEEC expression of angiopoietin (Ang)-2 and HESC expression of vascular endothelial growth factor (VEGF), interleukin (IL)-8 and matrix metalloproteinases (MMPs) while inhibiting HESC Ang–1 expression. The resultant inflammation and aberrant angiogenic stimulus leads to vessel damage causing local bleeding. The later delivers circulating factor VII to HESC membrane bound tissue factor (TF) which activates factor Xa to ultimately generate thrombin. The later exacerbates HX/ROS effects on endometrial angiogenesis and inflammation. In turn, HX and LAPCs trigger HEEC apoptosis by a paracrine manner. In addition, LAPCs also enhance FKBP51 expression in HESCs to inhibit progesterone receptor (PR; NR3C3) and glucocorticoid receptor (GR; NR3C1)-mediated transcription. In turn, the resultant PR and/or GR-mediated functional withdrawal reduces progestin and glucocorticoid mediated inhibition of endometrial inflammation, aberrant angiogenesis, thus contributing to AUB.

The endometria of women receiving LAPCs contain enlarged thin-walled fragile microvessels that are irregularly distributed across the superficial layer, and display intermittent focal bleeding. We previously observed that LAPC administration results in reduced endometrial blood flow which promotes local hypoxia (HX) and reactive oxygen species (ROS) generation damaging microvascular endothelial cells and enhancing production of pro-angiogenic factors secreted from HESCs (Fig 7) [8]. Specifically, the endometria of women receiving LAPCs display elevated levels of VEGF, the primary mediator of angiogenesis (Fig 7) [14]. Although physiological VEGF levels promote angiogenesis, over-expression of VEGF produces abnormal blood vessels with increased permeability and reduced perivascular ECM [38]. Moreover, our *in situ* observations and studies using primary cultures of HESCs and HEECs demonstrate that progestins inhibit HESC-expressed Ang–1, a vessel-stabilizing factor, while HX enhances HEEC-expressed Ang–2, which promotes vessel branching and enhances endothelial cell permeability [10]. Another recent study demonstrates that MPA and ETO treated HESCs secrete factors that promote HEEC apoptosis (Fig 7) [39].

Like women, GPs exhibit spontaneous estrous cycling and hemochorial placentation [40–43] justifying previous use by our laboratory of GPs as a relevant animal model to study LAPC-mediated endometrial changes [44, 45]. Our previous observations that LAPC administration reduced endometrial blood flow and induced local HX-mediated ROS generation in women [10], taken together with similar observations in the LAPC-treated GP endometrium [45], prompted evaluation of FKBP51 expression in the endometrium of GPs following administration of MPA. Increased FBP51 expression in MPA treated GP endometria confirms the observations made on post-DMPA administrated human endometria and supports presence of similar MPA mediated molecular mechanism in regulation of FKBP51 expression in both species.

A recent study [46] demonstrated that GR mediates anti-inflammatory action of MPA in cervical epithelial cells. Given the predominant expression of the GR in primary endocervical epithelial cells, this GR-mediated action may be essential in discriminating between the effects on inflammation caused by different progestins and P4. In support of the existence of functional cross-talk between the GR and LAPCs, Tomasicchio et al. [47] found that induction of apoptosis in primary CD4 (+) T-cells by MPA, but not P4 and suggested that progestin component of LAPCs may promote susceptibility to HIV–1. Complementing the conclusions drawn by these studies [46, 47], the current finding that MPA reduces basal IL-1β mRNA levels in HESCs is consistent with an anti-inflammatory action of MPA. Furthermore, reversal of this anti-inflammatory action of MPA by FKBP51 overexpression in HESCs supports our hypothesis that FKBP51 promotes functional P4 and/or GC withdrawal. In view of the microarray results in Tables 2–4 indicating that both MPA and ETO downregulate thrombin receptor expression, the finding that FKBP51 overexpression exacerbates thrombin induced IL-1β expression in the presence of progestin is even more striking.

In conclusion, results obtained from the current study reveal that MPA and ETO alter the expression of significantly greater numbers of genes than P4 in endometrial cells. Distinct from P4, MPA and ETO mediated regulation of numerous genes as exemplified by FKBP51 are linked to ligand dependent activation of the GR, but not the PR. These observations suggest the need for future studies that evaluate the critical role played by FKBP51 in mediating LAPC induced functional P4 and/or glucocorticoid withdrawal leading to AUB.

## Supporting Information

S1 Fig Glucocorticoid receptor (GR) response elements (GREs) in IGFBP2, ADAMTS1, F2RL2 and FKBP51 genes. IGFBP1, ADAMTS1, F2RL1 and FKBP51 genes are regulated by MPA and ETO in HESCs. GREs are identified by the MatInspector program. Specifically, GR binding sites represent the glucocorticoid responsive and related elements (V$GREF; purple box) and the negative glucocorticoid response elements (V$NGRE; blue box) matrix families. Arrows represent transcription start site.
(TIF)

S2 Fig Ingenuity Pathway Analysis (IPA) identifies glucocorticoid receptor (NR3C1) as a key upstream regulator in HESCs treated with either MPA or ETO. Upstream regulators of MPA and ETO induced differentially regulated genes in cultured HESCs using IPA analysis detected activation of NR3C1, the glucocorticoid receptor according to the gene list obtained from whole genome microarray analysis using Illumina HumanHT–12 v4 expression BeadChip kit analysis (n = 3/group). Orange squares indicate predicted increase in activity while blue squares indicate predicted decrease in activity. The circular network shows the upstream regulator in the center with its targets colored by the expression results (upregulated genes in red color, down-regulated genes in green color). Orange edge, leads to activation; yellow edge, inconsistent state; grey edge, effect not predicted. All gene symbols were abbreviated according to GENEBANK standard nomenclature.
(TIF)

S3 Fig Activation of AKT and ERK1/2 signaling pathways in HESC. Immunoblot analysis of phosphorylated (p-) and total (T-) levels of AKT and ERK1/2 MAPK in HESCs incubated with E_2_ (10^−8^ M) or E_2_ + P_4_ (10^−7^ M) or E_2_ + ETO (10^−7^ M) or E_2_ +MPA (10^−7^ M) for 24 hr (n = 3). β-actin was used as a loading control.
(TIF)
