# Supplementary figures and images for: Progestins Upregulate FKBP51 Expression in Human Endometrial Stromal Cells to Induce Functional Progesterone and Glucocorticoid Withdrawal: Implications for Contraceptive- Associated Abnormal Uterine Bleeding
Source: PLoS One. 2015 Oct 5;10(10):e0137855. doi: 10.1371/journal.pone.0137855 (PMC4593551; doi:10.1371/journal.pone.0137855)

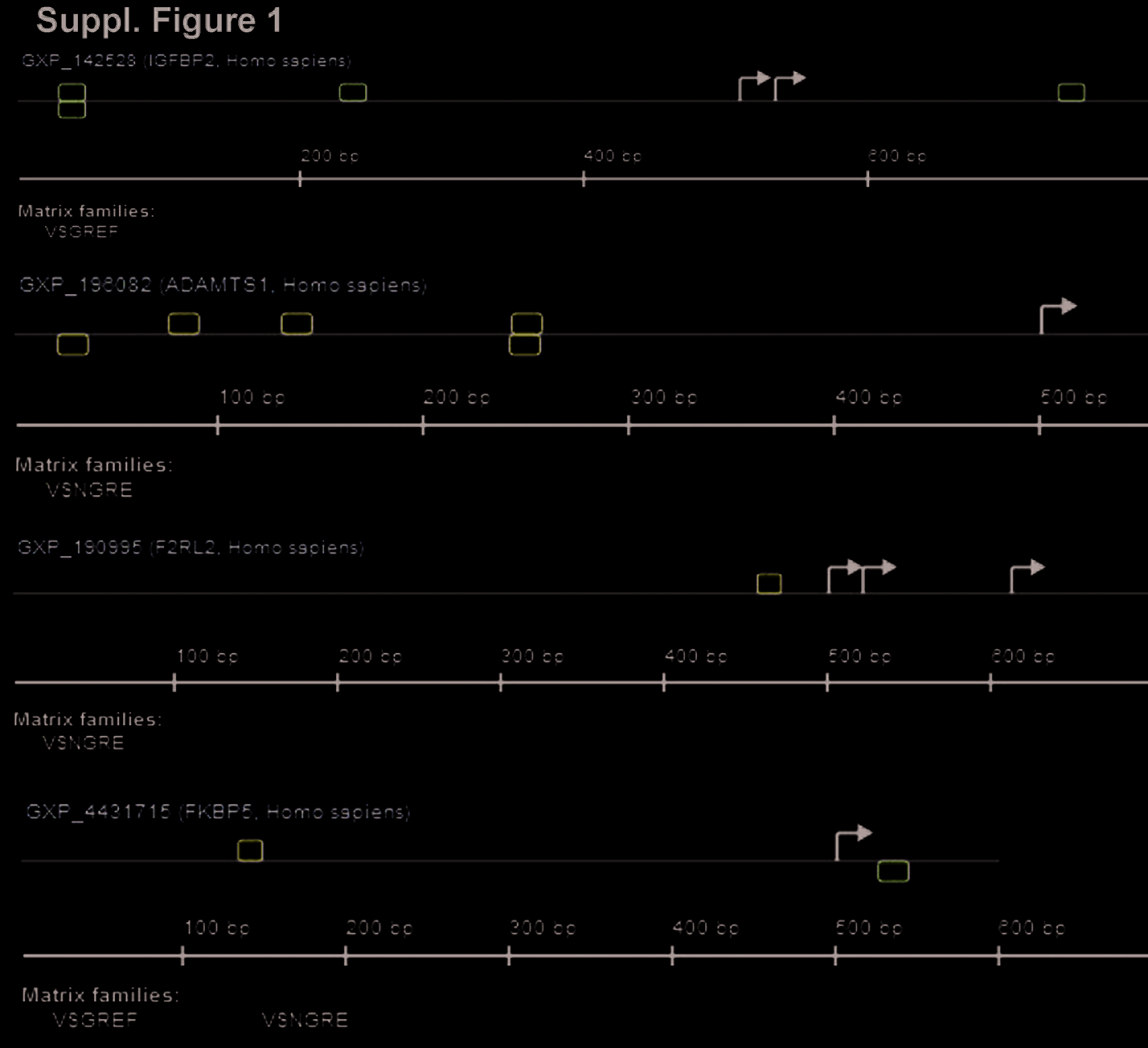

Supplement: S1 Fig — IGFBP1, ADAMTS1, F2RL1 and FKBP51 genes are regulated by MPA and ETO in HESCs. GREs are identified by the MatInspector program. Specifically, GR binding sites represent the glucocorticoid responsive and related elements (V$GREF; purple box) and the negative glucocorticoid response elements (V$NGRE; blue box) matrix families. Arrows represent transcription start site. (TIF) [file pone.0137855.s001.tif]

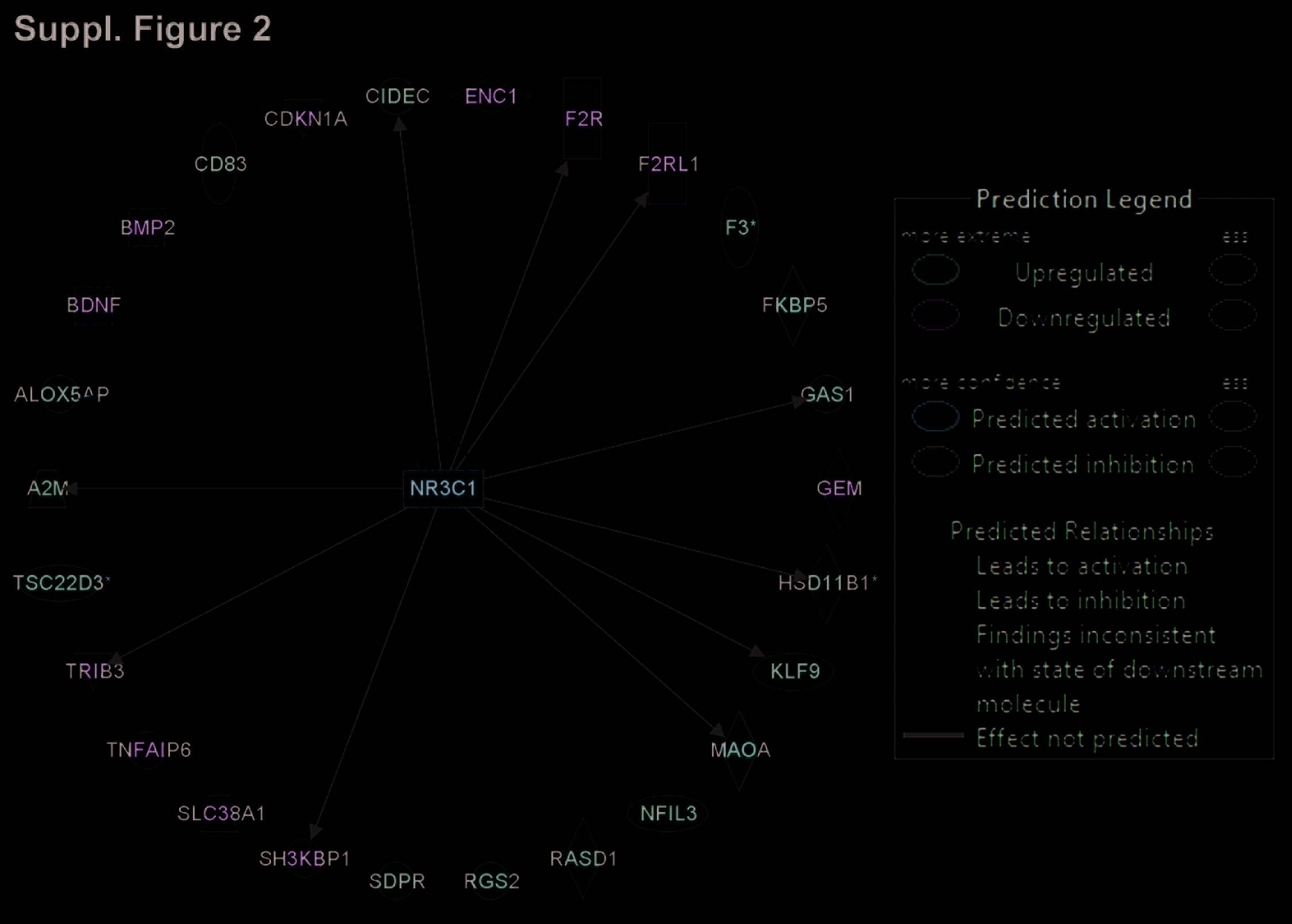

Supplement: S2 Fig — Upstream regulators of MPA and ETO induced differentially regulated genes in cultured HESCs using IPA analysis detected activation of NR3C1, the glucocorticoid receptor according to the gene list obtained from whole genome microarray analysis using Illumina HumanHT–12 v4 expression BeadChip kit analysis (n = 3/group). Orange squares indicate predicted increase in activity while blue squares indicate predicted decrease in activity. The circular network shows the upstream regulator in the center with its targets colored by the expression results (upregulated genes in red color, down-regulated genes in green color). Orange edge, leads to activation; yellow edge, inconsistent state; grey edge, effect not predicted. All gene symbols were abbreviated according to GENEBANK standard nomenclature. (TIF) [file pone.0137855.s002.tif]

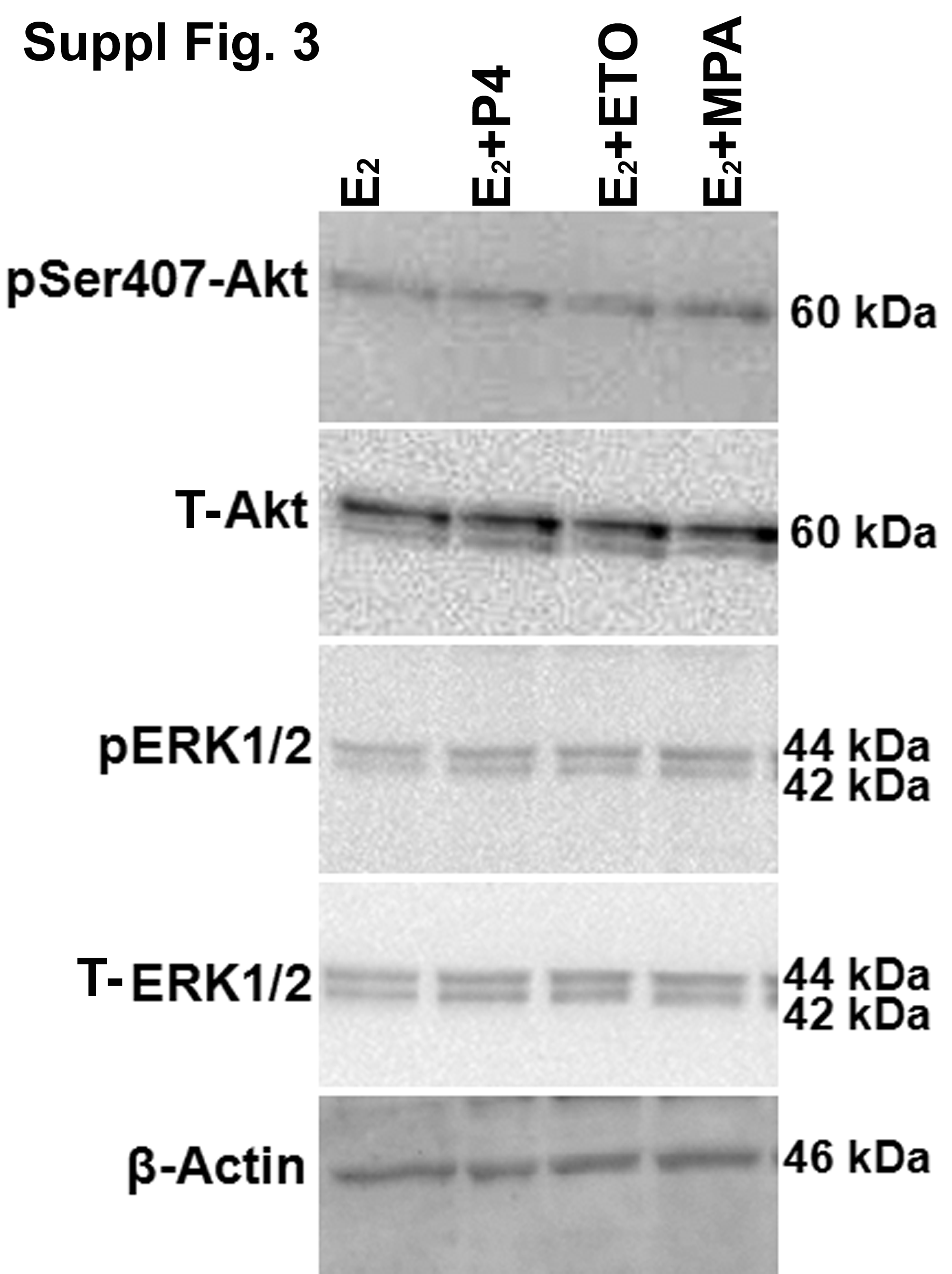

Supplement: S3 Fig — Immunoblot analysis of phosphorylated (p-) and total (T-) levels of AKT and ERK1/2 MAPK in HESCs incubated with E2 (10−8 M) or E2 + P4 (10−7 M) or E2 + ETO (10−7 M) or E2 +MPA (10−7 M) for 24 hr (n = 3). β-actin was used as a loading control. (TIF) [file pone.0137855.s003.tif]
